# Social Media Perceptions of Legacy-Making: A Qualitative Analysis

**DOI:** 10.1089/pmr.2020.0069

**Published:** 2020-12-14

**Authors:** Leonard L. Sokol, Sarah R. Jordan, Allison J. Applebaum, Joshua M. Hauser, Jodi Forlizzi, Moran Cerf, Hillary D. Lum

**Affiliations:** ^1^ The Ken and Ruth Davee Department of Neurology, Northwestern University Feinberg School of Medicine, Chicago, Illinois, USA.; ^2^ McGaw Bioethics Scholars Program, Center for Bioethics and Humanities, Northwestern University Feinberg School of Medicine, Chicago, Illinois, USA.; ^3^ Division of Geriatric Medicine, University of Colorado School of Medicine, Aurora, Colorado, USA.; ^4^ Department of Psychiatry and Behavioral Sciences, Memorial Sloan Kettering Cancer Center, New York, New York, USA.; ^5^ Center for Bioethics and Medical Humanities, Institute for Public Health and Medicine, Northwestern University Feinberg School of Medicine, Chicago, Illinois, USA.; ^6^ Section of Palliative Medicine, Department of Medicine, Northwestern University Feinberg School of Medicine, Chicago, Illinois, USA.; ^7^ Palliative Care Service, Jesse Brown VA Medical Center, Chicago, Illinois, USA.; ^8^ Human-Computer Interaction Institute, School of Computer Science, Carnegie Mellon University, Pittsburgh, Pennsylvania, USA.; ^9^ Kellogg School of Management, Northwestern University, Evanston, Illinois, USA.; ^10^ Eastern Colorado VA Geriatric Research Education and Clinical Center, Rocky Mountain Regional VA Medical Center, Aurora, Colorado, USA.

**Keywords:** digital legacy, meaning-centered therapy, memorialization, neuropalliative care, psychoneurology, social media

## Abstract

***Background:*** Individuals with life-limiting illnesses experience psychotherapeutic benefits of transmitting their life's history to loved ones; however, the scope and depth of what warrants preservation and who ought to undertake such activity remains less clear. Furthermore, individuals with conditions that afflict the brain face barriers regarding the timing and structure of such interventions. We analyzed data from an online social media forum to understand perceptions of legacy-making.

***Methods:*** This is a qualitative descriptive study of Slashdot, a social media website with a focus on science, technology, and politics. In August 2010, a Slashdot user inquired about a loved one with a life-limiting illness and asked for opinions on how to preserve the individual's memories. We conducted a content analysis of the individual comments related to digital legacy-making to identify common themes.

***Results:*** Slashdot users contributed 527 replies to the initial inquiry. Users often included bereaved individuals who offered input on the need to preserve information about a loved one, the modalities in which to preserve, and what type of content to preserve. Three key themes emerged related to legacy-making: (1) capture the individual's essence and avoid the minutia, (2) live for now to avoid prolonged suffering, and (3) recognize the equal benefits to all who memorialize.

***Conclusions:*** Users in a social media forum articulated the value of capturing their loved ones' essence for posterity, which many believed would help them to avoid prolonged grief. These findings have implications for the development and timing of personalized psychosocial interventions as well as informing application development of evidence-based digital legacy systems.

## Introduction

Individuals with life-limiting illnesses confront complex existential issues.^1–4^ Select interventions can effectively lessen these symptoms toward end of life, mostly within the cancer population.^2^ Meaning-centered psychotherapy (MCP), an efficacious psychotherapeutic intervention, focuses on four sources of meaning in life that can become resources for patients who experience various forms of suffering.^5^ One of these, the historical source of meaning, focuses on the legacy that we as humans are given and did not choose, the one we are currently creating, and the one we will give to others. Through the completion of the Legacy project, individuals take steps to reflect on the memorable experiences of life. Dignity therapy (DT) is another evidence-based intervention that emphasizes legacy-making using an exercise to create a legacy document.^2^

Expanding upon the conceptual foundations of legacy-making, several initiatives have pioneered digital approaches in tandem with the growth of digital data.^6–9^ However, limited evidence currently exists on the breadth and depth of what might be part of these systems and who ought to participate. With the advent of digital legacy-making, social media has become an apt platform to assess the nature of the illness experience.^10,11^ Since data collected on various social media are also a part of one's legacy, and as systems grow in number, this creates yet another fragmented view of an individual's life, and palliative care studies now commonly leverage blogs, forums, and networking sites for mixed-methods research.^12^ Slashdot, a social media forum, allows international users from around the world to discuss science, technology, and politics.^13^ Through querying public-domain search systems, we identified an online thread where an individual shared that a loved one had an estimated one to two years of life remaining and elicited suggestions on the utility of memorialization.^14^ This study aims to understand the perceptions of legacy-making for individuals with life-limiting illnesses, as discussed on a social media forum.

## Methods

### Context

In August 2010, a social media user sought suggestions on Slashdot on preserving memories surrounding a loved one.^14^ Over a month, the forum had ongoing replies and input, behaving similar to a virtual focus group. Users could reply to the original user or another user's comment. The data were public, and freely available for viewing. No demographic data were available for users, and the project team did not contact any user, per accepted protocols.^15^ Although the social media users were anonymous, this has not been a drawback in human–computer interaction studies.^9,15^ Past reports indicate that users of Slashdot represent those who are technologically inclined and have advanced levels of education.^13,16^ The Institutional Review Board at Northwestern reviewed our project and determined this study to be nonhuman subject research.

### Data analysis

We conducted a qualitative content analysis study to address key themes related to three inquiries: (1) Do individuals desire to create a legacy document? (2) If so, what deserves preservation? and (3) Who ought to undertake the activity?^17^ Our approach to exploring attitudes surrounding digital legacy systems related to death and dying was informed by Forlizzi and colleagues (human–computer interaction).^6,9,18^ The goal was to identify emerging themes related to this topic from the social media data, as we and others have done.^6–8,11^^,^^19^

We imported social media users' comments and replies into ATLAS.Ti (Version 8.4.24.0). Two authors (L.L.S. and S.R.J.) independently conducted the analysis as follows^20^: First, the coders read a user-generated comment many times to identify both the (1) manifest and (2) latent content. Second, during that process, the coders met and developed a codebook of nine different parent codes, reflective of our three key inquiries.^21^ The coders utilized a combined inductive and deductive approach^21^ to both identify nuances within comments based on the three questions and to allow for inductive exploration of emerging ideas. Third, coders independently applied the codebook to the dataset and generated preliminary reflections on the dataset in a “memoing” process. Fourth, coders discussed their memos and reviewed the coded dataset to ensure the codes were used consistently across the data to ensure reliability. To promote trustworthiness, the coders maintained detailed meeting notes from each coding session and collectively built consensus of emerging key themes as they related to the data until thematic saturation was achieved, where no new themes emerged.

## Results

The original social media user's inquiry received 527 comments. Most user comments analyzed from the online forum included perspectives of individuals who had either experienced a personal loss or knew someone else who had. Three key themes emerged related to the perceptions of legacy-making from social media: capturing the individual's essence and avoiding the minutia, living for now to avoid prolonged suffering, and recognizing equal benefits to all who memorialize. Table 1 highlights these themes and offers suggestions from Slashdot users.

**Table 1.** Themes and Suggestions for Legacy-Making from Users

Themes	Suggestions from online forum users^a^
Theme 1: capture the individual's essence and avoid the minutia	Describe the person, the character, and how they interact in different contexts
	Focus on the content; the medium is less critical; be sure to capture the individual's relationships
	Have the individual describe the significant milestones of life
	How the individual's personality came to bear during challenging times
	Focus not on particulars of the past or the future, but the philosophical aspects derived from those times
	Do not make a “shrine” and pray over it
Theme 2: live for now to avoid prolonged suffering	Do spend quality time with the individual and not be mesmerized with documenting as the individual will not be desired to be framed as he or she approaches end of life
	The attempt at capturing everything may cause harm and interfere with the natural grieving process; be cognizant of that
	Choose just select things to remember; too much and it can become overwhelming
	The recording of all parts of the individual's life will impede the natural process of life moving forward
	A balance exists between spending time and preserving memories; a disproportionate focus on the latter may lead to remorse
Theme 3: recognize the equal benefits to all who memorialize	Include loved ones and friends in the production of the legacy-making
	Have the first-degree relatives actively seek to perform actions/collect artifacts that can help in the memorialization
	The spouse of the individual with a life-limiting illness should collect photos and annotate each with a story to share with other loved ones
	Encourage that the focus should emanate from the individual and his or her thoughts and milestones
	Have siblings join and share memories about the individual with a life-limiting illness

^a^ Suggestions are paraphrased from social media user comments to preserve anonymity.

### Theme 1: Capture the individual's essence and avoid the minutia

As a loved one approaches end of life, users discussed the value of capturing the individual's personhood. Most users gave credence to seeing the “big picture” and sketching who the individual truly was—even well before the diagnosis of a life-limiting illness. There was a preponderance of opinions that valued (1) the memorialization of past, current, or future (anticipated) milestones; (2) the person's character and personality; (3) the response to philosophical questions; and (4) the relationships with others. Although users valued these concepts, the modality, be it a physical (e.g., collection of past letters, clothing, or scents) or digital medium (e.g., blog), was less critical so long as it was able to achieve these aims and capture the authenticity of the individual. Users eschewed the notion of recording the banalities of life—or constructing a “shrine.” Most perceived the idea of encoding the minutia as causing harm, rather than good, as it would have little value for review in the future. Overall, there was a reluctance to record interminable amounts of audio or video that showcased a play-by-play of the individual's life.

### Theme 2: Live for now to avoid prolonged suffering

Users emphasized the importance of living in the moment with the dying, as this was a natural way to create memories that would supersede death and curtail future suffering. Furthermore, some users felt that preservation of all things would complicate the grieving process, especially the notion of “hoarding” memories near the end of life. Thus, many issued a caveat about the senseless gathering of tidbits from a variety of mediums, including e-mails and text messages, and instead underscored the priority to be present and make preservation a secondary concern.

### Theme 3: Recognize the equal benefits to all who memorialize

Reciprocal psychosocial opportunities might exist—no matter who spearheads the activity of memorialization. Users recognized that creating a legacy might have benefits for the legacy creator and the legacy “readers.” For example, some reasoned that an individual with a life-limiting illness ought to explore topics that matter. The individual may use it as a reminder of a purposeful life as death nears. Friends or loved ones may also value viewing this not only as of the individual's death nears but also in the months after that. Conversely, if friends or loved ones lead collective efforts on the memorialization of the individual's essence, this may provide similar reciprocal psychosocial benefits. The individual with a life-limiting illness may also review the artifact to provide a sense of a life that had an impact on others.

## Discussion

The exploration and management of existential distress of individuals with life-limiting illnesses and their families remain an integral component of palliative medicine.^1,2^ Recently, digital legacy systems have emerged that aim to preserve memories of loved ones.^6,7,9^ At the same time, social media systems continue to grow in number; these capture fragmented data about an individual, leaving a different kind of legacy once one has passed.

Our findings described the value of legacy-making, what to preserve, and who should participate based on a social media forum that was similar to a virtual focus group. We found that users eschewed the construction of a shrine and instead sought conceptual preservation of the individual, primarily focusing on relationships, life philosophy, personality/character, and milestones. This finding supports the process of digital legacy-making as incorporating aspects of psychotherapeutic interventions such as MCP and DT.^2^ However, our findings are in contrast with some digital legacy systems that seek to immortalize the decedent, such as Eterni.me and Eter9.^6^ Indeed, the theme of “live for now to avoid prolonged suffering,” suggests that the creation of an electronic shrine may have unintended side effects.^6,22^ Thus, although legacy-making is a beneficial act, our findings suggest that it must be balanced or treated secondarily to the importance of being in the moment during the end-of-life experience.

An intriguing finding from our analysis is the agnostic attitude regarding who should undertake preservation. Although clinicians of different backgrounds can help facilitate the creation of a legacy document or project^2^ our data also suggest that benefits are possible if loved ones or friends spearhead the legacy-making. We see this practice akin to “saying goodbye” or reflective of a “pre-death” eulogy. To our knowledge, we are unaware of an evidence-based intervention that proposes the legacy-making to emanate from the reflections of loved ones and friends of the dying person.

Our analysis is not without limitations. First, the online forum likely comprised educated individuals with interests in technology, thus individuals with technology barriers (“digital divide”) are unlikely to have contributed.^13,16^ Second, we did not systematically recruit users, and the discussion occurred nearly a decade ago. Indeed, discussions in the 2020s may instead focus on “digital graveyards” where individuals' online footprints (i.e., their social media accounts) become a living testament to their lives after death. Third, most users did not seem to have a life-limiting illness, and thus perspectives of individuals with life-limiting illnesses are likely under-represented. Fourth, we had no demographic data to base our findings; our data may over-represent or under-represent certain groups. Fifth, although the online venue does make it easier to study these comments before the advent of today's digital age, it does pose certain drawbacks, as the site does not prohibit multiple submissions or allow for the public to examine how many unique comments from a given user were submitted. Therefore, we could not identify whether unique comments came from a repeat user.

Future work should study the value of digital legacy-making among actual patients and caregivers. Research should explore timing, structure, and adaptation of interventions to different populations.^4^ Studies should include diverse or vulnerable patient populations, including those with neurodegenerative illnesses, such as Parkinson or Huntington disease, who may have deficits in motor, attention, cognitive, language, and emotional functions over years, rather than weeks or months, compared with individuals with cancer.^4^ New avenues should enable friends and loved ones the opportunity to spearhead the legacy-making and to distribute the artifact to the dying and others. Finally, efforts should prioritize the inclusion of diverse groups, including under-represented minorities and those of lower socioeconomic status, who may not have access to uniform health care services.

In conclusion, we explored the value of legacy-making, such as what deserves preservation, and who ought to initiate the activity. Our results have implications on the timing and structure of interventions that aim to address existential distress, such as the use of digital legacy systems. Fields such as psychoneurology and neuropalliative care may use these findings to guide the adaptation of legacy interventions and the development of new ones.^4^

## Abbreviations Used

DT: dignity therapy
MCP: meaning-centered psychotherapy

## References

[1] Lemay K, Wilson K: Treatment of existential distress in life threatening illness: A review of manualized intervention. Clin Psychol Rev 2008;28:472–493 17804130 10.1016/j.cpr.2007.07.013

[2] Saracino RM, Rosenfeld B, Breitbart W, Chochinov HM: Psychotherapy at the end of life. Am J Bioeth 2019;19:19–28 10.1080/15265161.2019.1674552 PMC6986450 31746703

[3] Sokol LL, Young MJ, Paparian J, et al.: Advance care planning in Parkinson's disease: Ethical challenges and future directions. NPJ Park Dis 2019;5:24 10.1038/s41531-019-0098-0 PMC6874532 31799376

[4] Sokol LL, Lum HD, Creutzfeldt CJ, et al.: Meaning and dignity therapies for psychoneurology in neuropalliative care: A vision for the future. J Palliat Med 2020;23:1155–1156 32877285 10.1089/jpm.2020.0129 PMC7469693

[5] Cohen SR, Mount BM, Strobel MG, Bui F: The McGill Quality of Life Questionnaire: A measure of quality of life appropriate for people with advanced disease. A preliminary study of validity and acceptability. Palliat Med 1995;9:207–219 7582177 10.1177/026921639500900306

[6] Gulotta R, Gerritsen DB, Kelliher A, Forlizzi J: Engaging with death online. In: Proceedings of the 2016 ACM Conference on Designing Interactive Systems - DIs'16, 736–748, ACM Press, 2016. DOI: 10.1145/2901790.2901802

[7] Gulotta R, Kelliher A, Forlizzi J: Digital systems and the experience of legacy. In: Proceedings of the 2017 Conference on Designing Interactive Systems - DIs'17, 663–674, ACM Press, 2017. DOI: 10.1145/3064663.3064731

[8] Gulotta R, Odom W, Forlizzi J, Faste H: Digital artifacts as legacy. In: Proceedings of the SIGCHI Conference on Human Factors in Computing Systems - CHI’13, 1813, ACM Press, 2013. DOI: 10.1145/2470654.2466240

[9] Gulotta R, Odom W, Faste H, Forlizzi J: Legacy in the age of the internet. In: Proceedings of the 2014 conference on Designing interactive systems - DIs'14, 975–984, ACM Press, 2014. DOI: 10.1145/2598510.2598579

[10] Keim-Malpass J, Adelstein K, Kavalieratos D: Legacy making through illness blogs: Online spaces for young adults approaching the end-of-life. J Adolesc Young Adult Oncol 2015;4:209–212 26697270 10.1089/jayao.2015.0003 PMC4684647

[11] Cutshall NR, Kwan BM, Salmi L, Lum HD: “It makes people uneasy, but it's necessary. #BTSM”: Using Twitter to explore advance care planning among brain tumor stakeholders. J Palliat Med 2020;23:121–124 31170019 10.1089/jpm.2019.0077 PMC6931910

[12] Hopewell-Kelly N, Baillie J, Sivell S, et al: Palliative care research centre's move into social media: Constructing a framework for ethical research, a consensus paper. BMJ Support Palliat Care 2019;9:219–224 10.1136/bmjspcare-2015-000889 PMC6582818 26823291

[13] Bruns A: Stuff that matters: Slashdot and the emergence of open news. In: Proceedings of the 4th Association of Internet Researchers Conference 2003 1–19 (Association of Internet Researchers, 2003)

[14] Preserving memories of a loved one? https://ask.slashdot.org/story/10/08/14/2122257/preserving-memories-of-a-loved-one (Last accessed 122, 2020)

[15] Kraut R, Olson J, Banaji M, et al.: Psychological research online: Report of board of scientific affairs' advisory group on the conduct of research on the internet. Am Psychol 2004;59:105–117 14992637 10.1037/0003-066X.59.2.105

[16] Lampe CA: Ratings use in an online discussion system: The Slashdot case (Doctoral dissertation). https://deepblue.lib.umich.edu/bitstream/handle/2027.42/39369/lampe_diss_revised.pdf?sequence=2 (Last accessed 122, 2020)

[17] Vaismoradi M, Turunen H, Bondas T: Content analysis and thematic analysis: Implications for conducting a qualitative descriptive study. Nurs Heal Sci 2013;15:398–405 10.1111/nhs.12048 23480423

[18] Hsieh HF, Shannon SE: Three approaches to qualitative content analysis. Qual Health Res 2005;15:1277–1288 16204405 10.1177/1049732305276687

[19] Salmi L, Lum HD, Hayden A, et al.: Stakeholder engagement in research on quality of life and palliative care for brain tumors: A qualitative analysis of\# BTSM and\# HPM tweet chats. Neuro-Oncol Pract 2020; DOI: 10.1093/nop/npaa043 PMC7716141 33304602

[20] Creswell JW, Poth CN: Qualitative Inquiry and Research Design: Choosing Among Five Approaches. Thousand Oaks, CA: Sage Publications, 2016

[21] Fereday J, Muir-Cochrane E: Demonstrating rigor using thematic analysis: A hybrid approach of inductive and deductive coding and theme development. Int J Qual Methods 2006;5:80–92

[22] Savin-Baden M, Mason-Robbie V: Digital Afterlife: Death Matters in a Digital Age. Boca Raton, FL: CRC Press, 2020

